# 2-Methyl­anilinium dihydrogen phosphate–phospho­ric acid (1/1)

**DOI:** 10.1107/S1600536809014536

**Published:** 2009-04-30

**Authors:** Hamed Khemiri, Samah Akriche, Mohamed Rzaigui

**Affiliations:** a Laboratoire de Chimie des Matériaux, Faculté des Sciences de Bizerte, 7021 Zarzouna Bizerte, Tunisia

## Abstract

In the title compound, C_7_H_10_N^+^·H_2_PO_4_^−^·H_3_PO_4_, there is a clear distinction between the P—O/P=O and P—OH bond lengths. In the crystal, the H_2_PO_4_^−^ anions and H_3_PO_4_ mol­ecules are linked by O—H⋯O hydrogen bonds, leading to layers propagating in the *bc* plane. The organic cations are located between these layers and inter­act with them by way of N—H⋯O hydrogen bonds.

## Related literature

For related structures, see: Akriche & Rzaigui (2000 ▶); Zaccaro *et al.* (1996 ▶). For background, see: Desiraju (1995 ▶).
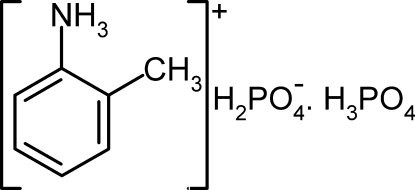


## Experimental

### 

#### Crystal data

C_7_H_10_N^+^·H_2_PO_4_^−^·H_3_PO_4_
*M* *_r_* = 303.14
Monoclinic, 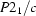

*a* = 10.8769 (10) Å
*b* = 7.938 (4) Å
*c* = 15.302 (3) Å
β = 91.57 (2)°
*V* = 1320.7 (7) Å^3^
*Z* = 4
Ag *K*α radiation
μ = 0.19 mm^−1^
*T* = 298 K
0.37 × 0.31 × 0.25 mm

#### Data collection

Enraf–Nonius CAD-4 diffractometer
Absorption correction: none
5416 measured reflections
5250 independent reflections
4134 reflections with *I* > 2σ(*I*)
*R*_int_ = 0.013
2 standard reflections frequency: 120 min intensity decay: 18%

#### Refinement

*R*[*F* ^2^ > 2σ(*F* ^2^)] = 0.033
*wR*(*F* ^2^) = 0.092
*S* = 1.08
5250 reflections
170 parameters
H-atom parameters constrained
Δρ_max_ = 0.30 e Å^−3^
Δρ_min_ = −0.41 e Å^−3^

### 

Data collection: *CAD-4 EXPRESS* (Enraf–Nonius, 1994 ▶); cell refinement: *CAD-4 EXPRESS*; data reduction: *XCAD4* (Harms & Wocadlo, 1995 ▶); program(s) used to solve structure: *SHELXS97* (Sheldrick, 2008 ▶); program(s) used to refine structure: *SHELXL97* (Sheldrick, 2008 ▶); molecular graphics: *ORTEP-3* (Farrugia, 1997 ▶) and *DIAMOND* Brandenburg (2005); software used to prepare material for publication: *WinGX* (Farrugia, 1999 ▶).

## Supplementary Material

Crystal structure: contains datablocks I, global. DOI: 10.1107/S1600536809014536/hb2955sup1.cif

Structure factors: contains datablocks I. DOI: 10.1107/S1600536809014536/hb2955Isup2.hkl

Additional supplementary materials:  crystallographic information; 3D view; checkCIF report

## Figures and Tables

**Table 1:** Selected bond lengths (Å)

P1—O3	1.4964 (9)
P1—O4	1.5092 (10)
P1—O2	1.5571 (9)
P1—O1	1.5707 (9)
P2—O8	1.4942 (9)
P2—O5	1.5422 (10)
P2—O7	1.5445 (10)
P2—O6	1.5493 (10)

**Table 2:** Hydrogen-bond geometry (Å, °)

*D*—H⋯*A*	*D*—H	H⋯*A*	*D*⋯*A*	*D*—H⋯*A*
O1—H1⋯O4^i^	0.82	1.83	2.6483 (16)	178
O2—H2⋯O8^i^	0.82	1.80	2.6132 (13)	170
O5—H5⋯O3	0.82	1.72	2.5351 (15)	176
O6—H6⋯O8^ii^	0.82	1.81	2.6223 (16)	170
O7—H7⋯O4^iii^	0.82	1.69	2.5109 (13)	177
N1—H1*A*⋯O1^i^	0.89	2.08	2.9627 (16)	172
N1—H1*B*⋯O3	0.89	1.91	2.7808 (19)	164
N1—H1*C*⋯O7^iv^	0.89	2.18	3.0086 (15)	154

Symmetry codes: (i) 
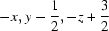
; (ii) 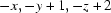; (iii) 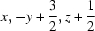; (iv) 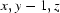.

**Fig. 1.**
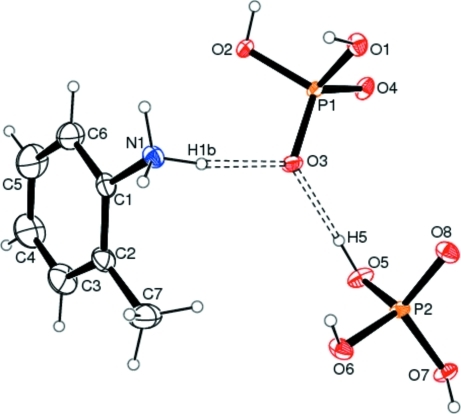
The molecular structure of (I) with displacement ellipsoids drawn at the 30% probability level. H atoms are represented as small spheres of arbitrary radius. Hydrogen bonds are represented as dashed lines.

**Fig. 2.**
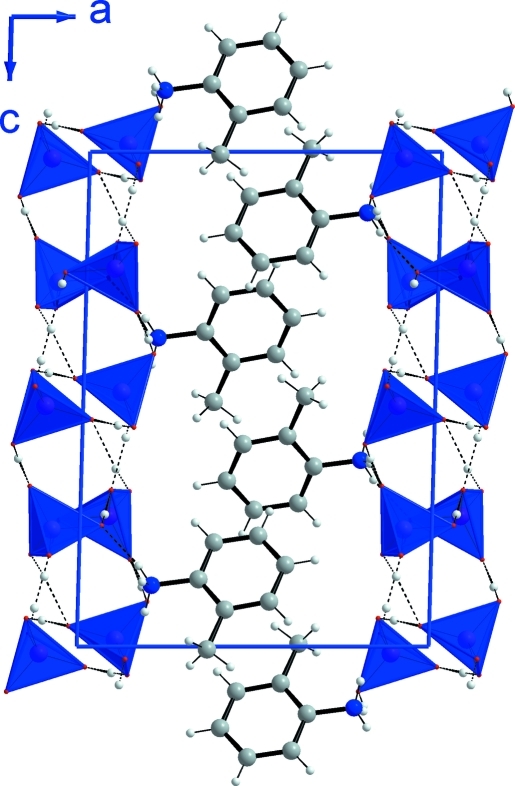
DIAMOND (Brandenburg, 2005) Projection of (I) along the b axis.

## supplementary crystallographic information

### Comment

Organic cation phosphates have been intensively studied due to their many uses in various fields such as biomolecular sciences, catalysts and nonlinear optics (e.g. Desiraju, 1995). Nevertheless, a bibliographical study on the organic monophosphates, and especially on the adduct monophosphate reveals that this kind of compounds are relatively very rare if compared with another types of phosphates (Zaccaro *et al.*, 1996).

In the atomic arrangement of the title compound (I), the asymmetric unit consists of three fundamentals entities, the H_2_PO_4-_ anion, the H_3_PO_4_ molecule and the organic cation C_7_H_10_N_+_ (Fig. 1). A view of the structure projected along the b direction (Fig. 2) shows that the inorganic entities are organized in layers developed around the *bc* plane. The organic cations are arranged in opposite direction along the *a* axis in the interlayer spacing to neutralize the negative charge of the inorganic layers. Inside each layer the H_2_PO_4-_ anions form an inorganic chains parallel to b direction and situated at *Z* = 1/4 and *Z* = 3/4. The H_3_PO_4_ molecules are associated by strong hydrogen bonds to form a dimmer of formula [H_6_P_2_O_8_] centred at (0 1/2 0) and (0 0 1/2). The both entities are interconnected together *via* hydrogen bonds to form inorganic layer parallel to the *bc* plane (Fig. 2). In the two crystallographically independent phosphate groups, the P—O bonds are shorter than P—OH bonds (Table 1). The average values of P—O distances and O—P—O angles are 1,533 Å, 109,44° and 1,533 Å, 109,38°, respectively for P(1)O_4_ and P(2)O_4_ tetrahedra. These configurations are comparable to that observed elsewhere (Zaccaro *et al.*,  1996). The organic and inorganic species establish between them two types of hydrogen bonds. The first one is O—H···O, involving short contacts with H···O lengths ranging between 1,69 - 1,83 Å, connects the H_2_PO_4-_ and H_3_PO_4_ entities to develop the inorganic layer parallel to *bc* plane. The second type is N—H···O, with H···O distances ranging from 1,91 Å to 2,18 Å, links the organic cations to the phosphoric layer. The pattern of hydrogen bonds participate with the electrostatic and van Der Waals interactions to the cohesion of the network. The atoms C1, C2, C3, C4, C5 and C6 of the anilinium ring of the title compound are coplanar and they form a conjugated plane with average deviation of 0.0013 Å. The C—C distances ranging from 1.374 (2) to 1.496 (3) Å agree with those observed in literature (Akriche & Rzaigui 2000).

### Experimental

A solution of orthophosphoric acid (0.50 mmol in 30 ml of water) was added drop by drop to an ethanolic solution of 2-methylaniline (2.336 mmol in 5 ml). The so-obtained solution was slowly evaporated at room temperature, until colourless prisms of (I) formed.

### Refinement

The H atoms were fixed geometrically and treated as riding with C—H = 0.93Å, N—H = 0.89 Å and O—H = 0.82 Å with *U*_iso_(H) = 1.2 *U*_eq_(carrier).

### Crystal data

**Table:**

C_7_H_10_N^+^·H_2_PO_4_^−^·H_3_PO_4_	*F*(000) = 632
*M**_r_* = 303.14	*D*_x_ = 1.525 Mg m^−^^3^
Monoclinic, *P*2_1_/*c*	Ag *K*α radiation, λ = 0.56085 Å
Hall symbol: -P 2ybc	Cell parameters from 25 reflections
*a* = 10.8769 (10) Å	θ = 8–12°
*b* = 7.938 (4) Å	µ = 0.19 mm^−^^1^
*c* = 15.302 (3) Å	*T* = 298 K
β = 91.57 (2)°	Prism, colorless
*V* = 1320.7 (7) Å^3^	0.37 × 0.31 × 0.25 mm
*Z* = 4	

### Data collection

**Table:**

Enraf–Nonius CAD-4 diffractometer	θ_max_ = 26.0°, θ_min_ = 2.1°
Radiation source: fine-focus sealed tube	*h* = −16→16
Nonprofiled ω scans	*k* = 0→12
5416 measured reflections	*l* = 0→23
5250 independent reflections	2 standard reflections every 120 min
4134 reflections with *I* > 2σ(*I*)	intensity decay: 18%
*R*_int_ = 0.013	

### Refinement

**Table:**

Refinement on *F*^2^	Primary atom site location: structure-invariant direct methods
Least-squares matrix: full	Secondary atom site location: difference Fourier map
*R*[*F*^2^ > 2σ(*F*^2^)] = 0.033	Hydrogen site location: inferred from neighbouring sites
*wR*(*F*^2^) = 0.092	H-atom parameters constrained
*S* = 1.08	*w* = 1/[σ^2^(*F*_o_^2^) + (0.0493*P*)^2^ + 0.181*P*] where *P* = (*F*_o_^2^ + 2*F*_c_^2^)/3
5250 reflections	(Δ/σ)_max_ = 0.001
170 parameters	Δρ_max_ = 0.30 e Å^−^^3^
0 restraints	Δρ_min_ = −0.41 e Å^−^^3^

### Special details

**Table:**

Geometry. All e.s.d.'s (except the e.s.d. in the dihedral angle between two l.s. planes) are estimated using the full covariance matrix. The cell e.s.d.'s are taken into account individually in the estimation of e.s.d.'s in distances, angles and torsion angles; correlations between e.s.d.'s in cell parameters are only used when they are defined by crystal symmetry. An approximate (isotropic) treatment of cell e.s.d.'s is used for estimating e.s.d.'s involving l.s. planes.
Refinement. Refinement of *F*^2^ against ALL reflections. The weighted *R*-factor *wR* and goodness of fit *S* are based on *F*^2^, conventional *R*-factors *R* are based on *F*, with *F* set to zero for negative *F*^2^. The threshold expression of *F*^2^ > σ(*F*^2^) is used only for calculating *R*-factors(gt) *etc*. and is not relevant to the choice of reflections for refinement. *R*-factors based on *F*^2^ are statistically about twice as large as those based on *F*, and *R*- factors based on ALL data will be even larger.

### Fractional atomic coordinates and isotropic or equivalent isotropic displacement parameters (Å^2^)

**Table:**

	*x*	*y*	*z*	*U*_iso_*/*U*_eq_	
P1	0.08064 (3)	0.43847 (3)	0.736601 (16)	0.02322 (6)	
P2	0.10749 (3)	0.71819 (3)	0.986049 (17)	0.02487 (7)	
O1	−0.06037 (8)	0.40074 (11)	0.74454 (6)	0.03314 (17)	
H1	−0.0695	0.3079	0.7670	0.050*	
O2	0.13778 (8)	0.28822 (10)	0.68641 (6)	0.03396 (18)	
H2	0.0974	0.2708	0.6413	0.051*	
O3	0.14542 (9)	0.44285 (11)	0.82411 (5)	0.03420 (18)	
O4	0.08566 (9)	0.59919 (10)	0.68413 (6)	0.03335 (18)	
O5	0.19612 (9)	0.70906 (12)	0.90923 (6)	0.0394 (2)	
H5	0.1806	0.6250	0.8798	0.059*	
O6	0.14555 (9)	0.58760 (11)	1.05701 (6)	0.0372 (2)	
H6	0.1003	0.5052	1.0530	0.056*	
O7	0.13831 (9)	0.89278 (10)	1.02544 (5)	0.03362 (18)	
H7	0.1232	0.8928	1.0776	0.050*	
O8	−0.02457 (8)	0.69700 (11)	0.95881 (6)	0.03445 (18)	
N1	0.20745 (9)	0.11752 (14)	0.87628 (7)	0.0355 (2)	
H1A	0.1698	0.0513	0.8370	0.053*	
H1B	0.1808	0.2228	0.8695	0.053*	
H1C	0.1910	0.0818	0.9298	0.053*	
C1	0.34057 (12)	0.11198 (18)	0.86394 (10)	0.0400 (3)	
C2	0.41777 (14)	0.1876 (2)	0.92552 (12)	0.0517 (4)	
C3	0.54313 (16)	0.1826 (3)	0.90905 (19)	0.0797 (7)	
H3C	0.5986	0.2305	0.9492	0.096*	
C4	0.58628 (19)	0.1090 (4)	0.8354 (2)	0.0930 (8)	
H4C	0.6702	0.1099	0.8252	0.112*	
C5	0.5070 (2)	0.0340 (4)	0.7763 (2)	0.0930 (8)	
H5C	0.5374	−0.0182	0.7269	0.112*	
C6	0.38195 (18)	0.0357 (3)	0.78970 (14)	0.0643 (5)	
H6C	0.3272	−0.0136	0.7496	0.077*	
C7	0.3703 (2)	0.2703 (4)	1.00570 (16)	0.0795 (7)	
H7A	0.3238	0.1902	1.0382	0.119*	
H7B	0.3185	0.3634	0.9890	0.119*	
H7C	0.4382	0.3104	1.0413	0.119*	

### Atomic displacement parameters (Å^2^)

**Table:**

	*U*^11^	*U*^22^	*U*^33^	*U*^12^	*U*^13^	*U*^23^
P1	0.03254 (13)	0.01806 (11)	0.01906 (11)	0.00111 (9)	0.00062 (9)	−0.00078 (8)
P2	0.03360 (14)	0.02046 (11)	0.02043 (11)	−0.00449 (9)	−0.00143 (9)	0.00068 (9)
O1	0.0333 (4)	0.0282 (4)	0.0379 (4)	−0.0007 (3)	0.0019 (3)	0.0069 (3)
O2	0.0416 (5)	0.0282 (4)	0.0317 (4)	0.0095 (3)	−0.0053 (3)	−0.0105 (3)
O3	0.0498 (5)	0.0286 (4)	0.0238 (4)	−0.0003 (3)	−0.0071 (3)	−0.0040 (3)
O4	0.0491 (5)	0.0222 (3)	0.0292 (4)	0.0023 (3)	0.0101 (3)	0.0047 (3)
O5	0.0494 (5)	0.0385 (5)	0.0309 (4)	−0.0126 (4)	0.0104 (4)	−0.0106 (4)
O6	0.0437 (5)	0.0286 (4)	0.0387 (5)	−0.0069 (3)	−0.0126 (4)	0.0104 (3)
O7	0.0533 (5)	0.0225 (3)	0.0252 (4)	−0.0075 (3)	0.0034 (3)	−0.0037 (3)
O8	0.0366 (4)	0.0299 (4)	0.0363 (4)	−0.0056 (3)	−0.0082 (3)	0.0101 (3)
N1	0.0315 (5)	0.0339 (5)	0.0409 (5)	−0.0036 (4)	−0.0042 (4)	0.0062 (4)
C1	0.0318 (5)	0.0355 (6)	0.0526 (8)	0.0023 (5)	−0.0013 (5)	0.0065 (6)
C2	0.0360 (6)	0.0522 (9)	0.0662 (10)	0.0007 (6)	−0.0108 (6)	−0.0003 (8)
C3	0.0317 (7)	0.0912 (16)	0.1154 (19)	0.0004 (9)	−0.0107 (10)	−0.0069 (14)
C4	0.0366 (8)	0.123 (2)	0.120 (2)	0.0120 (11)	0.0082 (11)	−0.0074 (19)
C5	0.0630 (13)	0.113 (2)	0.105 (2)	0.0177 (13)	0.0272 (13)	−0.0215 (17)
C6	0.0528 (9)	0.0726 (12)	0.0677 (11)	0.0044 (9)	0.0066 (8)	−0.0128 (10)
C7	0.0610 (12)	0.1016 (18)	0.0752 (14)	−0.0020 (11)	−0.0123 (10)	−0.0313 (13)

### Geometric parameters (Å, °)

**Table:**

P1—O3	1.4964 (9)	N1—H1C	0.8900
P1—O4	1.5092 (10)	C1—C6	1.374 (2)
P1—O2	1.5571 (9)	C1—C2	1.382 (2)
P1—O1	1.5707 (9)	C2—C3	1.394 (2)
P2—O8	1.4942 (9)	C2—C7	1.496 (3)
P2—O5	1.5422 (10)	C3—C4	1.365 (4)
P2—O7	1.5445 (10)	C3—H3C	0.9300
P2—O6	1.5493 (10)	C4—C5	1.368 (4)
O1—H1	0.8200	C4—H4C	0.9300
O2—H2	0.8200	C5—C6	1.381 (3)
O5—H5	0.8200	C5—H5C	0.9300
O6—H6	0.8200	C6—H6C	0.9300
O7—H7	0.8200	C7—H7A	0.9600
N1—C1	1.4659 (16)	C7—H7B	0.9600
N1—H1A	0.8900	C7—H7C	0.9600
N1—H1B	0.8900		
			
O3—P1—O4	115.69 (5)	C6—C1—N1	117.83 (14)
O3—P1—O2	105.94 (5)	C2—C1—N1	118.88 (14)
O4—P1—O2	111.37 (6)	C1—C2—C3	116.33 (18)
O3—P1—O1	111.84 (6)	C1—C2—C7	122.21 (15)
O4—P1—O1	104.60 (5)	C3—C2—C7	121.46 (18)
O2—P1—O1	107.21 (5)	C4—C3—C2	121.5 (2)
O8—P2—O5	113.47 (6)	C4—C3—H3C	119.3
O8—P2—O7	113.99 (6)	C2—C3—H3C	119.3
O5—P2—O7	101.89 (5)	C3—C4—C5	120.51 (19)
O8—P2—O6	110.89 (5)	C3—C4—H4C	119.7
O5—P2—O6	110.01 (6)	C5—C4—H4C	119.7
O7—P2—O6	106.02 (6)	C4—C5—C6	120.2 (2)
P1—O1—H1	109.5	C4—C5—H5C	119.9
P1—O2—H2	109.5	C6—C5—H5C	119.9
P2—O5—H5	109.5	C1—C6—C5	118.3 (2)
P2—O6—H6	109.5	C1—C6—H6C	120.9
P2—O7—H7	109.5	C5—C6—H6C	120.9
C1—N1—H1A	109.5	C2—C7—H7A	109.5
C1—N1—H1B	109.5	C2—C7—H7B	109.5
H1A—N1—H1B	109.5	H7A—C7—H7B	109.5
C1—N1—H1C	109.5	C2—C7—H7C	109.5
H1A—N1—H1C	109.5	H7A—C7—H7C	109.5
H1B—N1—H1C	109.5	H7B—C7—H7C	109.5
C6—C1—C2	123.26 (15)		
			
C6—C1—C2—C3	−0.3 (3)	C2—C3—C4—C5	−1.6 (5)
N1—C1—C2—C3	−178.19 (16)	C3—C4—C5—C6	1.6 (5)
C6—C1—C2—C7	179.8 (2)	C2—C1—C6—C5	0.3 (3)
N1—C1—C2—C7	1.9 (3)	N1—C1—C6—C5	178.2 (2)
C1—C2—C3—C4	0.9 (3)	C4—C5—C6—C1	−0.9 (4)
C7—C2—C3—C4	−179.1 (3)		

### Hydrogen-bond geometry (Å, °)

**Table:**

*D*—H···*A*	*D*—H	H···*A*	*D*···*A*	*D*—H···*A*
O1—H1···O4^i^	0.82	1.83	2.6483 (16)	178
O2—H2···O8^i^	0.82	1.80	2.6132 (13)	170
O5—H5···O3	0.82	1.72	2.5351 (15)	176
O6—H6···O8^ii^	0.82	1.81	2.6223 (16)	170
O7—H7···O4^iii^	0.82	1.69	2.5109 (13)	177
N1—H1A···O1^i^	0.89	2.08	2.9627 (16)	172
N1—H1B···O3	0.89	1.91	2.7808 (19)	164
N1—H1C···O7^iv^	0.89	2.18	3.0086 (15)	154

Symmetry codes: (i) −*x*, *y*−1/2, −*z*+3/2; (ii) −*x*, −*y*+1, −*z*+2; (iii) *x*, −*y*+3/2, *z*+1/2; (iv) *x*, *y*−1, *z*.
